# Endothelial progenitor cell-derived extracellular vesicles: the world of potential prospects for the treatment of cardiovascular diseases

**DOI:** 10.1186/s13578-024-01255-z

**Published:** 2024-06-05

**Authors:** De-Xin Chen, Chuang-Hong Lu, Na Na, Rui-Xing Yin, Feng Huang

**Affiliations:** 1 Department of Cardiology & Guangxi Key Laboratory of Precision Medicine in Cardio-cerebrovascular Diseases Control and Prevention & Guangxi Clinical Research Center for Cardio-cerebrovascular Diseases, The First Affiliated Hospital of Guangxi Medical University, No. 6 Shuangyong Road, Nanning, 530021 Guangxi China; 2 Department of Neuroscience, Scripps Research Institute, No.10550 North Torrey Pines Road, La Jolla, San Diego, CA 92037 USA

**Keywords:** Endothelial progenitor cells, Extracellular vesicles, Cardiovascular diseases, Therapeutic prospects

## Abstract

Cardiovascular diseases (CVDs) have emerged as a predominant threat to human health, surpassing the incidence and mortality rates of neoplastic diseases. Extracellular vesicles (EVs) serve as vital mediators in intercellular communication and material exchange. Endothelial progenitor cells (EPCs), recognized as precursors of vascular endothelial cells (ECs), have garnered considerable attention in recent years due to the potential therapeutic value of their derived extracellular vesicles (EPC-EVs) in the context of CVDs. This comprehensive review systematically explores the origins, characteristics, and functions of EPCs, alongside the classification, properties, biogenesis, and extraction techniques of EVs, with particular emphasis on their protective roles in CVDs. Additionally, we delve into the essential bioactive components of EPC-EVs, including microRNAs, long non-coding RNAs, and proteins, analyzing their beneficial effects in promoting angiogenesis, anti-inflammatory and anti-oxidant activities, anti-fibrosis, anti-apoptosis, and myocardial regeneration. Furthermore, this review comprehensively investigates the therapeutic potential of EPC-EVs across various CVDs, encompassing acute myocardial infarction, myocardial ischemia–reperfusion injury, atherosclerosis, non-ischemic cardiomyopathies, and diabetic cardiovascular disease. Lastly, we summarize the potential challenges associated with the clinical application of EPC-EVs and outline future directions, aiming to offer a valuable resource for both theoretical insights and practical applications of EPC-EVs in managing CVDs.

## Introduction

With the continuous growth of the global population and the intensification of aging, the incidence of cardiovascular diseases (CVDs) has exhibited a rapid increase. According to statistics, the number of CVDs patients has nearly doubled, from 271 million in 1990 to 523 million in 2019 [1]. CVDs not only have a high incidence rate but also pose extremely serious threats to human function and survival, causing up to 15 million deaths globally each year, making them the leading cause of human mortality [2]. Given this dire situation, researchers have conducted in-depth studies on the treatment methods for CVDs, with stem cell therapy receiving particular attention due to its unique advantages. Stem cells, with their remarkable self-renewal ability, low immunogenicity, and powerful proliferative potential, have demonstrated significant potential in the treatment of CVDs [3]. Endothelial progenitor cells (EPCs), a subset of stem cells, offer promise in CVDs treatment due to their ability to self-renew and differentiate into endothelial cells (ECs). Notably, Jimenez-Quevedo et al. demonstrated improved cardiac function in refractory angina pectoris patients through EPCs injection, showcasing the potential of stem cells in treating ischemic heart disease [4]. However, the clinical application of stem cells or progenitor cells is not without challenges. Issues such as the risk of embolism, ethical controversies, targeting difficulties, and the temporary preservation of cell biological functions have limited their widespread use in the treatment of CVDs [5]. Hence, there's a pressing need for a cell-free treatment method that circumvents these limitations.

In recent years, the study of extracellular vesicles (EVs) has provided new ideas for the cell-free treatment of CVDs. EVs are small membrane vesicles released by cells into the extracellular matrix and are widely present in various bodily fluids and cell supernatants. These tiny vesicles carry a rich array of cytokines, bioactive substances, proteins, and non-coding RNAs (ncRNAs), playing crucial roles in cell-to-cell communication, migration, angiogenesis, and cell growth [6–8]. Importantly, EVs can protect their internal active substances from interference and degradation by the external environment, thereby enhancing therapeutic effects.

Endothelial progenitor cell-derived extracellular vesicles (EPC-EVs) have garnered significant attention due to their unique biological properties. The therapeutic impact of EPC-EVs on ischemic tissues and organs surpasses the endothelial differentiation of EPCs [9]. EPC-EVs possess anti-inflammatory, anti-apoptotic, angiogenic, and tissue regenerative functions, making them prominent protectors in various CVDs such as acute myocardial infarction, myocardial ischemia–reperfusion injury, atherosclerosis, and diabetic cardiovascular disease [10, 11]. This review will comprehensively explore the application of EPC-EVs in the treatment of CVDs and provide an outlook for their future development prospects, aiming to provide new ideas and methods for advancing the field of CVDs treatment.

## EPCs

### Origin and characteristics

In 1997, Asahara T initially demonstrated the existence of precursor cells with the ability to differentiate into vascular ECs in the circulating peripheral blood, which they named EPCs [12]. Hur et al. [13] classified EPCs into two types based on the duration of culture: early EPCs and late EPCs. Early EPCs, which were spindle-shaped, reached their maximum growth at 2–3 weeks and disappeared after 4 weeks. Cobblestone-like EPCs emerge during the later stages, manifesting around 2 to 3 weeks, experiencing exponential growth from 4 to 8 weeks, and persisting for a maximum of 12 weeks. Due to their origin from individual nucleated cells, initial EPCs lack purity and are combined with numerous single nucleated cells, resulting in high expression of monocyte markers cluster differentiation (CD)14 and CD45, moderate expression of ECs marker CD31, and low expression of hematopoietic stem cell marker CD34 [14, 15]. Endothelial colony forming cells (ECFCs), also called as late-stage EPCs, exhibit a phenotype highly resembling that of ECs. ECFCs demonstrate significant expression of CD31, CD34, CD146, and vascular endothelial growth factor receptor 2 (VEGFR2), moderate expression of CD133 and VE-cadherin (CD144), and markers CD14 and CD45 must be negative [16–18]. Contrary to traditional beliefs, EPCs do not originate from the bone marrow but instead arise from an alternative niche within the vessel wall [19]. Moreover, recent studies have shown that they can also be obtained from peripheral tissues such as umbilical cord blood, heart, liver, lungs, adipose tissue, and vascular endothelium [20]. Those that are long-term or permanently residing in the tissues are referred to as tissue-resident EPCs. These cells are characterized by their high expression of markers such as CD31, CD117, CD105, CD157, and CD144 [21], Notably, CD117 is specific for tissue-resident EPCs. Carbonic Anhydrase 4 (Car4)^−^ high tissue-resident EPCs can stimulate lung repair after injury through VEGF-A signaling [22]. It should be emphasized that the interpretation of EPCs, surface indicators, and cultivation circumstances might differ in various experimental settings. However, EPCs are required to possess the ability to regenerate themselves, promote blood vessel growth, create an inner layer, and migrate consecutively to shape or merge with the circulatory system [23, 24]. EPCs from various origins are outlined in Table 1, providing a summary of their surface markers, culture media, and identification methods.

**Table 1:** Surface markers, media and identification methods for different sources of EPCs

Origins	Surface Markers (Positive)	Surface markers (negative)	Culture medium	Identification methods	References
Bone marrow (early EPCs)	CD14 CD31 CD45	CD34 CD133	EGM-2MV	Flow cytometry immunofluorescence staining	[14, 15]
Bone marrow (ECFCs)	CD31 CD34 CD133 CD146 VEGFR2 VE-Cadherin	CD45 CD14 CD11b	EGM-2MV	Flow cytometry immunofluorescence staining	[16, 17]
Cord blood	CD31 CD105 CD146 CD141 CD144 VEGFR2 vWF	CD14 CD45	EGM-2MV	Flow cytometry immunofluorescence staining	[18]
Peripheral blood	CD31 CD34 CD133 VE-Cadherin VEGFR-2	CD14 CD45	EGM-2MV	Flow cytometry immunofluorescence staining	[20]
Fatty tissue	CD133 vWF VEGFR-2	CD14 CD45	EGM-2MV	Flow cytometry immunofluorescence staining	[20]
Tissue resident (heart, liver, lungs, peripheral vasculature, etc.)	CD31 CD117 CD105 CD157 VE-Cadherin	CD14 CD45 CD140a CD140b CD326	EGM-2MV	Flow cytometry immunofluorescence staining	[21]
Car4^+^ tissue-reident EPCs	Car4 VEGF-A CD105	CD14 CD45	EGM-2MV	Flow cytometry immunofluorescence staining	[22]

(*CD* cluster differentiation, *ECFCs* endothelial colony forming cells, *VEGFR2* vascular endothelial growth factor receptor-2, *VE-Cadherin* vascular endothelial cadherin, *vWF* von willebrand factor, *ICAM-2* intercellular adhesion mlecule 2, *Car4* carbonic anhydrase 4)

### Function

As precursor cells of ECs, EPCs have the ability to release different substances that encourage the growth, movement, and creation of blood vessels, including vascular endothelial growth factor (VEGF) and fibroblast growth factor **(**FGF). These substances play a role in the development of new blood vessels and the modification of existing ones [25, 26]. After a heart attack, ECFCs promote the growth of new blood vessels near the damaged heart tissue, demonstrating their strong ability to stimulate angiogenesis [27]. Furthermore, when exposed to physiological or pathological stimuli like injury, lack of blood flow, and oxygen deficiency [28–31], EPCs have the ability to relocate from the bone marrow to the injured area and transform into ECs. This process aids in the rejuvenation and restoration of the tissue's vascular endothelium, ultimately leading to the recovery of vascular function and the preservation of a regular blood flow to ischemic tissues. The angiogenic potential of EPCs was showcased above all cases. Additionally, EPCs have the ability to contribute to the control of immune response and reduction of inflammatory damage by functioning as antigen-presenting cells [32–34].Besides, EPCs can inhibit platelet activation, aggregation, adhesion, and thrombosis by binding platelets via CD62P and upregulating the secretion of cyclooxygenase-2 and prostacyclin [35].

## EVs

### Classification and characterization

EVs are intricate lipid bilayer structures actively secreted by cells, exhibiting a wide range of sizes spanning from 40 nm to 2000 nm. Conventionally, These EVs can be further classified into distinct subtypes: exosomes (50–150 nm), microvesicles (100–1000 nm), and apoptotic bodies (500–2000 nm), each delineated by its specific size range. Under the lens of cryo-electron microscopy, exosomes manifest a rounded configuration encased within bilobed membranes, presenting an almost natural state. Conversely, traditional transmission electron microscopy submerging them in methylcellulose induces exosomes to undergo contraction, thereby giving rise to an artificial cup-like morphology, while microvesicles and apoptotic bodies are morphologically variable [36]. Moreover, the density of these three distinct EV subtypes varies, and they often showcase a repertoire of characteristic biomolecules [37]. These may include tetratransmembrane proteins (CD9, CD63, and CD81), specific stress proteins (HSP70, HSP90), constituents of the endosomal sorting complex required for transport (Tsg101, Alix), as well as proteins involved in membrane fusion (Rabs, ARF6), and signaling proteins. In terms of perspective classification, protein markers like CD63, CD81, Tsg101, and Alix are predominantly found within exosomes, whereas selectins, integrin, and CD40 are associated with microvesicles, and markers for apoptotic bodies encompass Caspase 3 and histones [38]. In addition, EVs encompass a diverse array of bioactive cargo, encompassing DNA, mRNA, ncRNA, proteins, lipids, and beyond. Particularly noteworthy is the composition of apoptotic bodies, which encapsulate a plethora of cellular constituents, including histones, DNA, organelles, and membranes/cytoplasm [39, 40]. Functionally, EVs predominantly serve as conduits for intercellular information exchange and material transfer, thereby playing pivotal roles in various physiological and pathological processes.

### Biogenesis

**Fig. 1:**
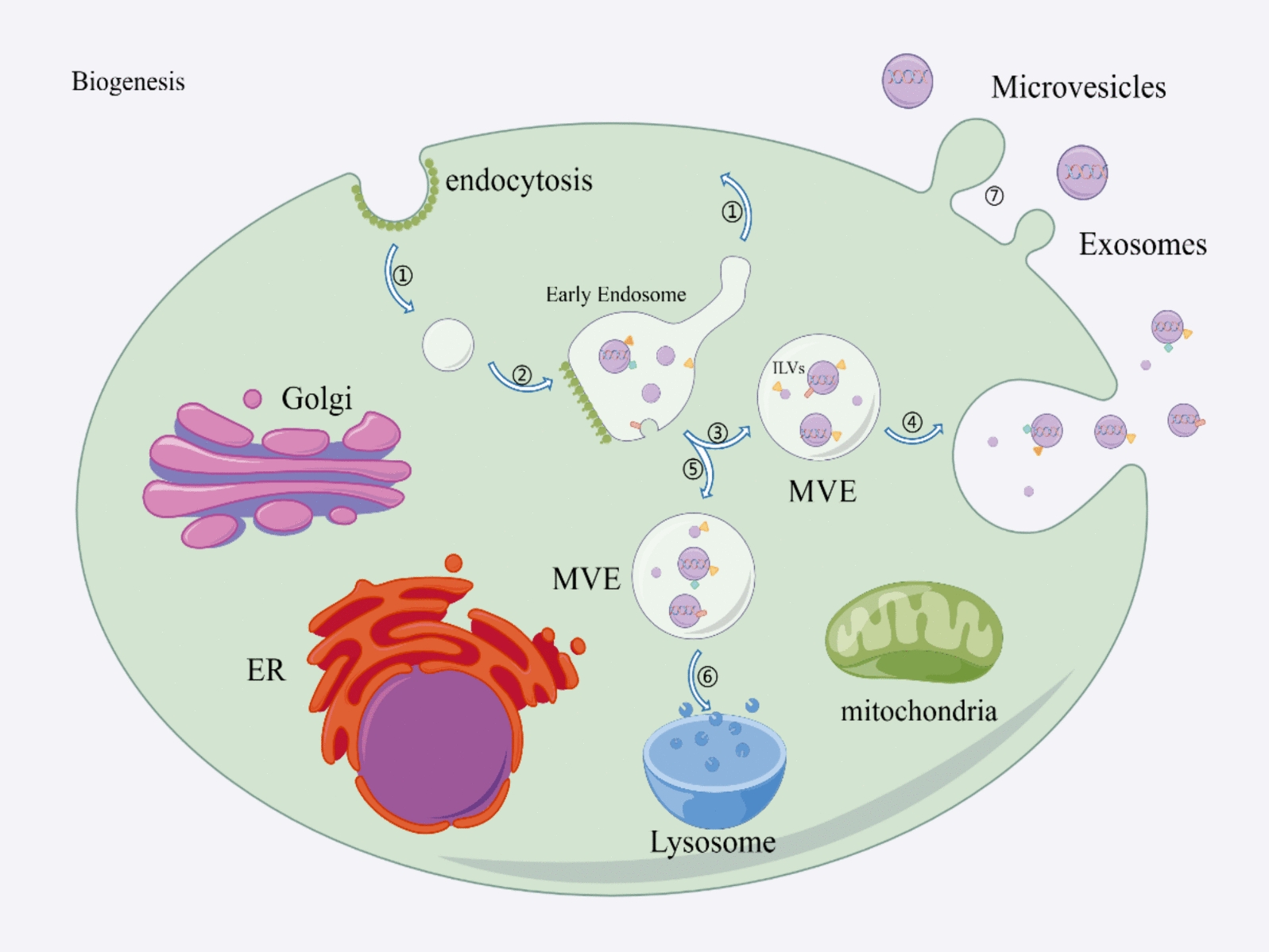
Biogenesis of EVs. Processes 1–6 show the biogenesis of exosomes and microvesicles. The process of phagocytosis or recycling facilitates the inward budding of the cell membrane, leading to the formation of early endosomes as the cytosol envelops the phagocytosed cargo (1–2). Early endosomes develop to form MVEs, which contain many ILVs. ILVs phagocytose proteins, lipids, and nucleic acids, which are subsequently released extracellularly through fusion with the cell membrane or reverse cytotransportation, thereby generating exosomes. (3–4). Certain MVEs do not undergo release into the extracellular milieu. Instead, they are transported to lysosomes for degradation (5–6). The cell membrane flows outward to bud to form microvesicles (7). (*EVs* extracellular vesicles, *MVE* multivesicular endosome, *ILV* intraluminal vesicle, *ER* endoplasmic reticulum, *Golgi* Golgi apparatus)

Endosomes are engulfed by the cell membrane through endocytosis, resulting in the formation of multivesicular body (MVB) in the cytoplasm. These MVBs contain numerous intraluminal vesicles (ILVs). The primary process of ILV formation is facilitated by the in vivo endosomal sorting complex required for transport (ESCRT) [41]. Additionally, ILVs can be transported and released through non-ESCRT-dependent tetratransmembrane proteins [42] and small GTPases of the Rab family [43, 44]. ILVs phagocytose proteins and nucleic acids, and MVBs release ILVs into the extracellular compartment by fusing with the cellular membrane. This release also occurs through retrograde cytocytosis approximately every 10 min, resulting in the formation of exosomes [45]. Microvesicles, in contrast to exosomes, are released at regular intervals, through the reorganization of the cytoplasmic framework and the movement of lipid layers. They are created by either merging with or releasing from the outer cell membranes [46]. The degradation of the plasma membrane-associated cytoskeleton is accompanied by Ca^2+^-dependent protein hydrolysis, which aids in the outgrowth process [47]. Moreover, it has been discovered that the activation of adenosine diphosphate-ribosylation factor 6 not only stimulates actin-dependent membrane shedding but also enhances the growth of microvesicles [48]. Apoptotic bodies are formed when cells undergo substantial alterations in the nucleus and cytoplasm as part of apoptosis, ultimately fragmenting into multiple disassemblies [49]. Figure 1 illustrates the unique biogenesis patterns of the three distinct EVs. The characteristics of exosomes, microvesicles, and apoptotic bodies are compared in Table 2.

**Table 2:** Comparison of three types of extracellular vesicles

	Exosomes	Microvesicles	Apoptotic bodies
Origin	MVB	Plasma membrane	Intracellular debris
Diameter(nm)	50–150	100–1000	500–2000
Density(g/ml)	1.13–1.18	1.16–1.19	1.16–1.28
Shape	Cup / circles	Variety	Variety
Formation method	Plasma membrane buds inward	Plasma membrane outgrowth	Plasma membrane outgrowth
Pathways	ESCRT-dependent tetratransmembrane protein-dependent Rab small GTPase- dependent	Ca^2+^-dependent ARF6-dependent	Apoptosis related pathways
Release interval	Ten more minutes	A few tenths of seconds	–
Protein markers	CD9 CD63 CD81 Tsg101 Alix	Selectins intergrin CD40	Caspase3 histones
Extraction Methods	Ultracentrifugation, Density Gradient Centrifugation	Ultracentrifugation, Field Flow Separation	Ultracentrifugation, Field Flow Separation
Identification Methods	WB NTA	WB NTA	WB NTA
Contents	DNA, mRNA, ncRNA, Proteins, lipids, etc	DNA, mRNA, ncRNA, Proteins, lipids, etc	Histone, DNA, Organelle and membrane/cytoplasmic components
Biological functions	Intracellular information transmission, extracellular material exchange	Intracellular information transmission, intercellular material exchange	Clearance of apoptotic cells, and regulation of pathological processes
Illustrations			

(*MVB* multivesicular body, *ESCRT* endosomal sorting complex required for transport, *GTP* guanosine triphosphate, *ARF6 ADP*-ribosylation factor 6, *Tsg* tumor susceptibility gene, *Alix* ALG-2-interacting protein X; *NTA* nanoparticle tracking analysis, *ncRNA* non-coding RNA)

### Manufacturing methods

Currently, there are numerous methods available for the isolation and purification of clinical-grade EVs for medical applications, including ultracentrifugation, density gradient centrifugation, filtration membrane separation, precipitation, and size exclusion chromatography, each with their own advantages and disadvantages [50]. Differential centrifugation, one of the most commonly used methods, employs centrifugal forces at various speeds to gradually remove cellular debris, large vesicles, and other impurities, ultimately leaving a precipitate of EVs. The key to this technique lies in selecting the appropriate rotational speeds and centrifugation durations to achieve optimal separation. While this method is relatively simple and cost-effective, suitable for large-scale EV production, it may be limited by the cost of extraction equipment, the influence of factors such as instrument quality and sample viscosity on extraction efficiency, and the inability to distinguish EVs from different cellular sources. Density gradient centrifugation involves the formation of a density gradient within a centrifuge tube, allowing EVs to deposit based on their density differences within the gradient. These vesicles are then collected through fractionation, often in combination with ultracentrifugation, to enhance purity [51]. However, this process is relatively complex and time-consuming, often requiring 16 h or more. Additionally, it can be influenced by sample properties and instrumental factors. Filtration membrane separation employs membranes with specific pore sizes to filter extracellular fluids, selectively retaining EVs based on their size. This straightforward technique is suitable for processing smaller sample volumes. However, the pore size of the membrane is crucial for effective extraction, as inappropriate membranes may lead to the loss or contamination of vesicles [52]. Precipitation methods involve the use of certain chemicals, such as polyethylene glycol, to interact with EVs and cause them to precipitate. The precipitated EVs are then collected through centrifugation or filtration. While this approach is simple and offers high recovery rates, it may introduce exogenous chemicals that could potentially affect subsequent experiments. Size exclusion chromatography (SEC) utilizes a porous stationary phase (typically a gel) to separate EVs based on their size. Larger vesicles cannot enter the pores of the gel and are thus eluted more quickly, while smaller vesicles penetrate the pores and remain within the gel for a longer duration, resulting in a delayed elution [53]. It is important to note that each method has its own scope of application and limitations. Therefore, in practical applications, it is necessary to select and optimize the methods based on specific needs, sample properties, and laboratory conditions. Additionally, to ensure the quality and purity of extracted EVs, it may be necessary to combine multiple methods for comprehensive extraction and purification.

### Cell-free RNAs and EV-derived RNA in CVDs risk stratification

RNA transportation is a complex process involving multiple molecules and cellular structures. Although EVs play a significant role in ncRNA transportation, the majority of ncRNA does not rely on EVs for transportation. Instead, they associate with ribonucleoproteins or larger complexes, such as Ago2 and nucleophosmin 1, to evade degradation by extracellular RNA enzymes [54]. In the circulatory system, free RNA can be transported through blood or other bodily fluids, potentially originating from processes like apoptosis, necrosis, or active secretion. While free RNA may be more abundant in quantity compared to RNA transported by EVs, they are susceptible to external influences and lack robust stability and protective mechanisms. The role of EVs in RNA transportation primarily lies in protecting and targeted delivery. EVs encapsulate RNA, creating a relatively stable environment that shields it from degradation and immune system attacks [55]. Additionally, EVs can target and deliver RNA to specific cells or tissues, enabling precise gene regulation or therapy [56]. In risk stratification for CVDs, the detection of free RNA may exhibit higher sensitivity and a broader application range. For instance, miR-208a demonstrates higher diagnostic sensitivity and specificity for AMI compared to cardiac troponin I (cTnI) [57]. miR-21 levels in the plasma of AMI patients are significantly elevated and correlate with cTnI and creatine kinase-MB (CK-MB)[58]. Moreover, the combination of miR-1 and miR-499 significantly improves diagnostic efficiency, surpassing cardiac troponin T (cTnT) [59]. The variety of ncRNA within EVs depends on the nature of the originating cells and surrounding environmental stimuli, rendering EV-derived RNA detection highly specific and targeted. Therefore, to comprehensively assess the application value of RNA in cardiovascular disease risk stratification, it is crucial to consider the characteristics and advantages of both free RNA and EV-derived RNA.

### Protective role of EVs in CVDs

While EVs were initially acknowledged solely as transporters of metabolic byproducts, they have recently been discovered to possess notable defensive properties in CVDs [60–64], particularly when released by stem/progenitor cells. Under hypoxic conditions, mesenchymal stem cells release EVs containing a high amount of miR-486-5p. These EVs activate matrix metalloproteinase 19-VEGFA signaling in fibroblasts, leading to the stimulation of post-infarction angiogenesis and cardiac repair [65]. Furthermore, Exosome (EXOs) derived from embryonic stem cells can improve adriamycin-induced cardiomyopathy by decreasing inflammation-induced cardiomyocytes (CMs) death and promoting a shift towards anti-inflammatory M2 macrophage polarization [66]. Additionally, EVs have demonstrated promising therapeutic potential in treating different CVDs. For instance, administering miR-21 enriched EVs locally can successfully reinstate heart function following an acute myocardial infarction (AMI) episode [67]. Moreover, studies have revealed that EXOs derived from mesenchymal stromal cells alleviates injury caused by myocardial ischemia–reperfusion injury (MIRI) by regulating macrophage polarization through miR-182 [68]. Likewise, EVs obtained from the serum of mice during exercise augmented the defensive impact of naturally occurring EVs against MIRI by activating ERK9 / 2 and HSP1 signaling pathways [69]. The above findings indicate that EVs could potentially enhance the restoration of cardiac function following AMI and MIRI. In addition, EVs derived from various types of cells including macrophages, ECs, and mesenchymal stem cells were found to improve atherosclerosis through microRNA or YRNA [70–74]. In 2018, Otani et al. conducted a study and found that EXOs derived from the plasma of normal rats by intraperitoneal injection could reduce blood pressure in hypertensive rats [75]. Studies also have demonstrated that EVs released by cardiosphere-derived cells have a positive impact on heart function in arrhythmogenic cardiomyopathy by reducing cardiac inflammation and inhibiting arrhythmogenesis [76]. In a model of heart failure caused by reduced blood flow, CMs released EVs containing miR-30d, which improved the negative changes in the heart's structure and function following heart failure. This improvement was achieved by acting on cardiac fibroblasts nearby, resulting in reduced expression of genes associated with fibrosis and inflammation [77]. Thus, EVs play an irreplaceable role in many CVDs, and fully exploiting their therapeutic value will contribute to clinical development.

## EPCs vs EPC-EVs

While both EPCs and EPC-EVs safeguard ECs against harm and stimulate angiogenesis, the beneficial impact of EPC-EVs on CVDs surpasses the direct involvement of EPCs [78]. In contrast to the surface indicators of EPCs, ALG-2 interacting protein X (ALIX), tumor susceptibility gene 101 (TSG101), CD9, and calnexin serve as the surface indicators of EPC-EVs [79]. Additionally, in contrast to EPCs, EPC-EVs offer the subsequent benefits: (1) Initial EPCs are not completely pure, exhibiting molecular characteristics linked to monocytes [80], and the introduction of EPCs through direct injection might stimulate cells with inflammatory capacity, potentially leading to life-threatening situations [81]. Furthermore, cell implantation therapy results in a reduction of over 90% in cell volume, and the rates of long-term cell implantation are exceedingly low [20]. However, EPC-EVs significantly improve CVDs by releasing bioactive substances like cytokines, growth factors, proteins, and ncRNAs into the surrounding tissues. This includes activating endogenous EPCs, forming neointima, and inhibiting CMs hypertrophy and apoptosis [82–84]. (2) EPC-EVs can act as a carrier for drug delivery. They retain membrane components more effectively, have a stronger ability to cross biological barriers, and exhibit good targeting capabilities [85–87]. (3) EPC-EVs demonstrate high biocompatibility, strong stability, and low immunogenicity [88, 89]. Allogeneic transplantation does not result in obvious rejection reactions [90]. (4) Compared to living cells, EPC-EVs have a longer shelf-life and can be transported and stored for extended periods [89]. (5) Packaging EPC-EVs with hydrogel or nanotechnology can significantly enhance therapeutic efficacy, which includes improving stability, targeting, and reducing loss rates [11, 91]. It is easier to be converted into clinical drug therapy [92]. To summarize, EPC-EVs offer greater benefits compared to EPCs alone when it comes to safeguarding ischemic tissues against harm. Therefore, forthcoming research should prioritize exploring the advantageous impacts of EPC-EVs.

## Contents of EPC-EVs

EPC-EVs comprise diverse bioactive compounds like microRNA (miRNA), long non-coding RNA (lncRNA), proteins, and nucleic acids. These substances have crucial functions in numerous physiological and pathological processes within the body, encompassing cell growth, programmed cell death, cellular specialization, and immune system regulation. Hence, examining the contents of EPC-EVs holds immense importance in understanding the development and management of CVDs.

### MiRNA of EPC-EVs

MiRNA, as a type of non-coding RNA, is about 22 nt long and plays a role in controlling gene expression by binding to target gene transcripts in a complementary manner. An increasing number of research studies have indicated that miRNAs in EPC-EVs have significant involvement in numerous pathophysiological processes, such as cellular growth and specialization, healing of tissues, formation of new blood vessels, as well as anti-inflammatory and antioxidant functions [93–95]. Among various miRNAs, miR-126 is the most abundant one that enhances the therapeutic effects of EPC-EVs in diseases. The enrichment of miR-126 potentiates the therapeutic benefits of EPC-EXOs in diabetic ischemic stroke by alleviating acute injury and promoting neural functional recovery [93]. Furthermore, EPC-EXOs downregulate SPRED1 and activate the Raf/ERK signaling cascade in a miR-126-dependent manner, thereby enhancing ECs proliferation, migration, and angiogenesis, which in turn induces bone regeneration in large bone defects[96]. Moderate exercise can enhance the protective effects of circulating EPC-EXOs on ECs against hypoxic injury in ischemic stroke through the miR-126/BDNF/TrkB/Akt pathway [97]. Additionally, miR-126-3p/5p in EPC-EXOs suppresses the inflammatory response triggered by high-mobility group 1 (HMGB1) and the permeability factor VEGFα, increases tight junction protein connections, and alleviates LPS-induced lung injury [95]. Moreover, miR-126-5p and 3p in EPC-EXOs separately inhibit LPS-induced HMGB1 and vascular cell adhesion molecule 1 (VCAM1) levels in human microvascular ECs (HMVBCs), thereby improving sepsis in mice [94]. Apart from miR-126, other enriched miRNAs also play pivotal roles in EPC-EVs. Studies have shown that EPC-EXOs improve endothelial dysfunction in diabetic atherosclerotic mice, which is associated with the top 10 upregulated miRNAs in EPC-EXOs, including miR-21a-5p, miR-222-3p, miR-221-3p, and miR-155-3p [98]. miR-222-3p in EPC-EXOs promotes M2 macrophage polarization and functional recovery in mice after spinal cord injury through the SOCS3/JAK2/STAT3 pathway [99]. miR-21-5p in EPC-EXOs inhibits the proliferation and anti-apoptosis of pulmonary artery smooth muscle cells in vitro and improves pulmonary hypertension by regulating the Mitofusin-2 and Ras-Raf-ERK1/2 signaling pathways [100]. EPC-EXOs deliver miR-21-5p to suppress Thrombospondin-1 expression, thereby promoting the repair of ECs in rats with balloon injury [101]. miR-210 loading can enhance the protective effects of EPC-EXOs against hypoxia/reoxygenation (H/R)-induced neuronal apoptosis, oxidative stress, and reduced viability [102].

Interventions can alter the expression levels and enhance the functions of miRNAs in EPC-EXOs. ACE-overexpressing EPC-EXOs (ACE-EPC-EXOs) inhibit cellular senescence, EC oxidative stress, apoptosis, and dysfunction, thereby improving brain neurovascular injury in elderly mice with ischemic stroke, through the activation of the miR-17-5p/PTEN/PI3K/Akt signaling pathway [103]. Additionally, ACE-EPCs-EXOs downregulate Nox2/ROS through miR-18a to alleviate H/R injury in senescent ECs [104]. Moreover, exercise protects N2a cells from hypertension-induced damage by improving mitochondrial function in EPC-EXOs, which may be associated with the increased levels of miR-27a in EPC-EXOs after exercise [105]. miR-133 is specifically sorted into H/R-induced EPC-EXOs through YBX-1 to enhance fibroblast angiogenesis and mesenchymal-endothelial transition (MEndoT) [84].

Late-stage ECFCs-derived EVs contain miRNAs that significantly improve EC function. miR-21-5p in ECFCs-derived exosomes regulates autophagy flux by suppressing SIPL1A2 to promote vascular endothelial repair and improve atherosclerosis [106]. Furthermore, ECFCs-derived EVs repair hypoxia-induced retinal damage in mice by promoting angiogenesis, which is associated with the enrichment of miR-451 and miR-486-5p in EVs [107]. Additionally, miRNAs also have certain improving effects on ferroptosis and apoptosis. EPC-EXOs transfer miRNA-30e-5p to regulate Erastin-induced ferroptosis in human umbilical vein ECs through the specific protein 1/AMPK axis [108]. EPC-EVs transfer miR-199a-3p to inhibit specific protein 1 (SP1), thereby suppressing ferroptosis in ECs and delaying the occurrence of atherosclerosis [109]. MiR-137 enhances the neuroprotective effects of EPC-EXOs on apoptosis and mitochondrial dysfunction in SH-SY5Y cells treated with oxyhemoglobin through the COX2/PGE2 pathway [110].

### lncRNA of EPC-EVs

LncRNA refers to a kind of ncRNA that has a size exceeding 200 nucleotides, and has significant functions in various life processes, including balancing gene expression, controlling epigenetic changes, managing cell division, and overseeing cellular specialization [111–113]. The utilization of lncRNA found in EPC-EVs provides significant advantages in disease treatment. It has been shown that hypoxic EPC-EVs enhanced cardioprotection by targeting miR-497 through lncRNA metastasis-associated lung adenocarcinoma transcript 1 (MALAT1) in a mouse myocardial infarction model [114]. Likewise, Cui and his colleagues found that lncRNA MALAT1 in EPC-EVs enhanced the recruitment and differentiation of osteoclast precursors to promote bone repair [115]. Further, it was found that lncRNA taurine upregulated gene 1 (TUG1) in EPC-EVs upregulated sirtuin 1 (SIRT1) to facilitate the polarization of M2 macrophages by competitively binding to miR-9-5p thereby ameliorating sepsis [116]. Table 3 summarizes the mechanisms of ncRNAs (miRNAs and lncRNAs) in EPC-EVs or EPC-EXOs in different diseases.

**Table 3:** The functional mechanisms of non-coding RNAs, specifically miRNAs and lncRNAs, within EPC-EVs (EXOs), in various pathological conditions

Non-coding small RNA	Disease/model	Mechanism/pathway	Function	References
miR-133	Primary fibroblasts	–	Increasing fibroblast angiogenesis and and MEndoT	[84]
miR-126	Diabetic ischemic stroke mice	–	Reducing acute injury and promoting neurological recovery	[93]
miR-126-3p/5p	LPS-induced lung injury	Upregulation of tight junction proteins	Reducing inflammation and improving lung damage	[95]
miR-126	Unilateral tibial distraction rats	SPRED1/Raf / ERK	Stimulating angiogenesis and accelerating bone regeneration	[96]
miR-126	Endothelial cells hypoxia/reoxygenation	BDNF/ TrkB/ Akt	Reducing inflammation,oxidative stress reactions and cell apoptosis	[97]
miR-126-3p/5p	Septic mice	HMGB1 /VCAM1	Reducing vascular leakage and improving sepsis	[94]
miR-21a-5p;miR-222-3p;miR-221-3p;miR-155-3p	Diabetic atherosclerotic mice	–	Improvement of endothelial dysfunction	[98]
miR-222-3p	Spinal Cord Injury mice	SOCS3/JAK2/STAT3	Promoting M2 macrophage polarization and repair after spinal cord injury	[99]
miR-21-5p	Pulmonary arterial hypertension	Mfn2/ Ras-Raf-ERK1/2	Promoting cell viability, proliferation and migration and attenuating apoptosis	[100]
miR-21-5p	Balloon injury rat	thrombospondin-1	Promoting the repair of ECs	[101]
miR-210	Neuronal hypoxia/reoxygenation	–	Attenuating neuronal apoptosis and oxidative stress	[102]
miR-17-5p	Aged ischemic stroke mice	PTEN/PI3K/Akt	Inhibition of ECs senescence, oxidative stress, apoptosis and dysfunction	[103]
miR-18a	ECs hypoxia/reoxygenation	Nox2/ROS	Attenuating endothelial cell apoptotic injury	[104]
miR-27a	Hypertensive mice	Mitochondria	Protection against N2a cell injury under hypertensive conditions	[105]
miR-21a-5p	Diabetic atherosclerotic mice	SIPA1L2	Reducing plaque formation and inflammatory factor production, improving endothelial dysfunction	[106]
miR-451/miR-486-5p	Mice with retinal damage	SIPL1A2	Protecting endothelial cells from damage and promoting angiogenesis	[107]
miR-30e-5p	HUVECs iron death	SP1-AMPK	Reducing endothelial cell damage	[108]
miR-199a-3p	Atherosclerosis mice	SP1	Inhibition of iron death and oxidative stress in endothelial cells	[109]
miR-137	Oxyhemoglobin-treated SH-SY5Y cells	COX2/PGE2	Inhibiting iron death and enhancing neuroprotection	[110]
MALAT1	Acute myocardial infarction mice	miR-497	Reducing fibrosis and improving angiogenesis and cardiomyocyte survival	[114]
MALAT1	Mouse Fracture Model	miR-124	Enhancing osteoclast formation and promoting bone repair	[115]
TUG1	Septic mice	miR-9-5p-SIRT1	Promoting macrophage polarization to M2 type and ameliorating sepsis	[116]

*SPRED1* sprouty-related, *EVH1* domain containing 1, *Raf* root abundant factor, *ERK* extracellular regulated protein kinases, *LPS* lipopolysaccharide, *HMGB1* high mobility group box 1, *VCAM1* vascular cell adhesion molecule 1, *MEndoT* mesenchymal-endothelial transition, *RUNX1* runt-related transcription factor 1, *SIPA1L2* signal induced proliferation related protein 1 like protein 2, *AMPK* Adenosine 5 ‘-monophosphate -activated protein kinase, *COX2* *cyclooxygenase-2*, *PGE2* prostaglandin E2, ELF5, *E74-like factor 5;* *AMI* acute myocardial infarction, *TUG1* taurine upregulated gene 1, *SIRT1*, sirtuin 1, *MALAT1* metastasis associated lung adenocarcinoma transcript 1

### Proteins in EPC-EVs

Although there is a lack of research on proteins in EPC-EVs, specific proteins including interleukins and enzymes have prominent roles in anti-inflammation and tissue repair. Yue et al. demonstrated that interleukin-10 (IL-10) knockout EPC-EVs significantly attenuated therapeutic efficacy in myocardial infarcted mice, by increasing infarcted area as well as reducing vascular regeneration after infarction, which was accomplished through enrichment of integrin-linked kinase (ILK) [117]. This suggests that IL-10 plays an important anti-inflammatory and reparative role in EPC-EVs. Similarly, diabetes impairs the reparative function of EPC-EVs in the ischemic heart through histone deacetylase-mediated downregulation of histone 3 lysine 9 acetylation in EPC-EVs [118]. Similarly, diabetes impairs the reparative function of EPC-EVs in the ischemic heart through histone deacetylase-mediated downregulation of histone 3 lysine 9 acetylation in EPC-EVs [119]. In another study, osteocalcin (OCN) overexpression in EPC-EXOs promotes angiogenesis by inhibiting G protein-coupled receptor family C group 6 member A (GPRC6A) expression [120]. In summary, EPC-EXOs that overexpress ACE have shown to inhibit cellular senescence, oxidative stress, apoptosis, and dysfunction in ECs, while activating the miR-17-5p/PTEN/PI3K/Akt signaling pathway. This mechanism has led to the amelioration of cerebral neurovascular injury in aged ischemic stroke mice [103]. These findings suggest that proteins contained within ECs hold therapeutic promise for CVDs.

## Role of EPC-EVs

Over the past few years, an increasing number of research studies have indicated that EPC-EVs have positive effects on CVDs, including promoting angiogenesis, facilitating tissue repair, preventing fibrosis and apoptosis, among others [121, 122]. The specific mechanism by which EPC-EVs play a role is controversial, and is summarized in detail in this paper.

### EPC-EVs and angiogenesis

EPC-EVs exhibit a significantly greater pro-angiogenic capacity compared to EPCs. They are delivered to ischemic myocardium via shearable hydrogels (STGs) to enhance peri-infarct myocardial angiogenesis and myocardial hemodynamics [11]. Additionally, delayed delivery of EPC-EVs by STGs significantly improves the duration of action and therapeutic efficacy [123]. These vesicles contain a myriad of contents, including RNAs, proteins, and lipids, each contributing to their pro-angiogenic effects through various mechanisms. Engineered EPC-EVs are enriched with miR-126a-3p and angiogenic factors such as VEGF, SDF-1, CXCR4, and eNOS. This enrichment significantly enhances hemodialysis, activating ECs and recruiting EPCs from the circulatory system to promote post-infarction angiogenesis [91]. Exosomes secreted by ECFCs containing microRNAs play a crucial role in ameliorating retinal ischemia and neurodegeneration by stabilizing the hypoxic vascular system, fostering blood vessel growth, and supporting nerve cells [89]. Hypoxia-treated EPC-EVs exhibit a more pronounced promotion of angiogenesis in ECs compared to EPC-EVs, attributed to the significant up-regulation of angiogenesis-associated miRNAs such as miR-155, miR-183, and miR-296 [101]. Moreover, EPC-EXOs facilitate neovascularization and mouse skin wound healing through the hsa_circ_0093884/miR-145/SIRT1 axis, ultimately enhancing ischemic hindlimb perfusion in mice [124]. Circulating EPC-EXOs enriched with miRNA-126, boosted by moderate exercise, safeguard ECs from hypoxic injury and foster angiogenesis [97]. Additionally, EPC-EXOs downregulate SPRED1 in a miR-126-dependent manner, activating the Raf/ERK signaling pathway to enhance EC proliferation, migration, and angiogenesis [96]. Conversely, oscillatory shear stress (OSS)-induced EPC-EXOs prompt endothelial mesenchymal transition (EndoMT) and hinder angiogenesis through the circ-1199/let-7 g-5p/HMGA2 signaling pathway [125]. osteocalcin (OCN) overexpression in EPC-EXOs promotes angiogenesis by inhibiting G protein-coupled receptor family C group 6 member A (GPRC6A) expression [120]. Wu and his colleagues discovered that EVs derived from cardiovascular precursor cells improved heart attack conditions by decreasing the death of CMs and stimulating the growth of blood vessels. Additionally, it was observed that this beneficial impact could be strengthened by cardiovascular precursor cells exposed to low oxygen levels [114]. Similarly, it has been shown that injecting hydrogel-loaded EPC-EVs into the ischemic myocardium significantly enhanced angiogenesis and improved cardiac hemodynamics [11]. Mathiyalagan et al. found that EPC-EXOs promotes angiogenesis by upregulating angiogenesis-related genes and improves blood flow in ischemic limbs of mice [82]. Furthermore, Zhang et al. revealed that activation of Erk1/2 signaling by EPC-EXOs from humans promoted angiogenesis and accelerated skin wound healing [126]. To summarize, EPC-EVs exhibit outstanding properties in promoting angiogenesis.

### EPC-EVs and anti-inflammatory and anti-oxidant

The inflammatory response triggers alterations in both intra- and extracellular environments, fostering heightened production of oxygen free radicals and other reactive oxidizing substances, thus inducing oxidative stress. At the same time, oxidative stress amplifies intracellular oxygen free radicals and reactive oxidizing substances, further fueling the inflammatory response. This reciprocal interaction prompts the infiltration of inflammatory cells and the release of inflammatory mediators [127, 128], and the anti-inflammatory and antioxidant effects of EPC-EVs can alleviate CVDs [129]. Inhibition of the nuclear factor kappa-B (NF-κB) signaling pathway and suppression of inflammatory cytokines like tumor necrosis factor-α (TNF-α) and IL-6 are achieved through the action of IL-10 found in EPC-EVs, leading to the attenuation of myocardial inflammatory responses [117]. Besides, EPC-EVs can interact with immune cells and fulfill an immunomodulatory function. The lncRNA TUG1 present in EPC-EVs can competitively attach to miR-5-9p, resulting in the up-regulation of SIRT1 expression and facilitating the polarization of M2-type macrophages, thereby exerting an anti-inflammatory effect [116]. Furthermore, EPC-EVs mitigate the harm caused by pro-inflammatory cytokines (CK) and complement protein C5a to glomerular ECs (GECs) by reducing oxidative stress. Additionally, they hinder leukocyte adhesion and alleviate inflammatory reactions by suppressing the expression of adhesion molecules (ICAM-1, VCAM-1, E-selectin) [130]. MiR-21-5p in EPC-EVs attenuated serum inflammatory response and oxidative stress by suppressing runt-related transcription factor 1(RUNX1) expression and attenuated sepsis-induced acute kidney injury [131]. In addition, serum deprivation of EPC-EVs also attenuated oxidative stress in ECs and reduced reactive oxygen species production [10]. EPC-EXOs with ACE2 overexpression provided protection to aged ECs against hypoxia/reoxygenation injury via the miR-18a/Nox2/ROS pathway. This protection was evidenced by a reduction in reactive oxygen species (ROS), an elevation in nitric oxide (NO) levels, and a decrease in the rate of apoptosis [104]. Additionally, ACE2-overexpressing EPC-EXOs demonstrated efficacy in ameliorating ischemic stroke in aged mice by mitigating cellular senescence, endothelial oxidative stress, apoptosis, and dysfunction through activation of the miR-17-5p/PTEN/PI3K/Akt signaling pathway [103]. Furthermore, loading of miR-210 enhanced the protective effects of EPC-EXOs against hypoxia/reoxygenation-induced neuronal apoptosis, oxidative stress, and reduced viability [102]. In summary, EPC-EVs can significantly attenuate the inflammatory response and oxidative stress, which is an important mechanism for EPC-EVs to alleviate CVDs.

### EPC-EVs and anti-fibrosis

The fibrotic process, a pathophysiological response marked by excessive collagen fiber accumulation in damaged tissues, not only disrupts tissue structure but also severely impairs function. In this context, EPC-EVs show promising anti-fibrotic capabilities by delivering encapsulated bioactive molecules, especially microRNAs (miRNAs), to finely regulate fibrosis. Studies have shown that EPC-EVs can reprogram resident renal cells using miRNAs, thereby preventing capillary thinning, glomerulosclerosis, and tubulointerstitial fibrosis, effectively ameliorating renal ischemia–reperfusion injury [132]. This reprogramming process involves miRNA-mediated regulation of target gene expression, impacting cell proliferation, differentiation, and function. In EPC-EXOs, upregulation of miRNAs like miR-218-5p and miR-363-3p promotes p53 expression, while downregulation of JMY expression aids in promoting mesenchymal-endothelial transition (MEndoT). This transformation process is crucial for inhibiting myocardial fibrosis, as it fosters EC regeneration and repair, thus halting fibrosis progression [133]. Understanding the regulatory role of these miRNAs elucidates the molecular mechanism of EPC-EVs in the anti-fibrotic process. Furthermore, specific interventions can enhance the anti-fibrotic capacity of EPC-EVs. For instance, hypoxia/reoxygenation interventions induce a high concentration of miR-133 in EPC-EXOs, thereby enhancing fibroblast angiogenic capacity and the MEndoT process [84]. This suggests that by mimicking environmental stimuli under physiological or pathological conditions, we can optimize the function of EPC-EVs and better tailor them to anti-fibrotic therapy needs. Additionally, the choice of delivery method significantly impacts the anti-fibrotic effect of EPC-EVs. A shear-thinning gel delivery system markedly improves fibrosis, reduces myocardial scar thickness, and enhances myocardial contractility two weeks after myocardial infarction compared to infusion of EPC-EVs alone [123]. This optimization of delivery not only improves the stability and distribution of EPC-EVs in vivo but also enhances their interactions with target cells, resulting in improved anti-fibrotic effects.

In summary, EPC-EVs regulate the fibrotic process through biologically active molecules such as miRNAs and enhance their anti-fibrotic capacity through specific interventions and optimization of delivery mode. Studying these mechanisms not only deepens our understanding of EPC-EVs in treating CVDs but also provides a strong theoretical basis and experimental foundation for developing new anti-fibrotic therapies.

### EPC-EVs and anti-apoptosis

CVDs occurrences like AMI and MIRI result in massive apoptosis of CMs and coronary artery ECs, and there is also massive CMs apoptosis and increased fibrosis in the end stage of heart failure. EPC-EVs can improve cardiac microangiogenesis by affecting ECs apoptosis, which will also rescue CMs that are about to be infarcted. Previous studies have demonstrated that depriving the serum of EPC-EVs effectively improves endothelial dysfunction and apoptosis caused by hypoxia/reoxygenation [10]. EPC-EXOs exhibit multifaceted reparative effects on ECs: they mitigate LPS-induced EC apoptotic damage and foster angiogenesis by modulating the Bcl2/Bax/Caspase-3 pathway. Additionally, they bolster endothelial function by facilitating the repair of ECs within the balloon injury area in rats [134]. Moreover, the abundance of miR-21-5p in EPC-EXOs exerts inhibitory effects on the Ras-Raf-ERK1/2 signaling pathway, dampening the proliferation and apoptosis of pulmonary artery smooth muscle cells in vitro by targeting the expression of Mitofusin-2 [100]. Furthermore, ACE-overexpressing EPC-EXOs demonstrate efficacy in ameliorating ischemic stroke in elderly mice by activating the miR-17-5p/PTEN/PI3K/Akt signaling pathway, thereby suppressing cell senescence, endothelial oxidative stress, apoptosis, and dysfunction [103]. Additionally, ACE-overexpressing EPC-EXOs alleviate hypoxia/reoxygenation-induced EC apoptotic damage by activating the miR-18a/Nox2/ROS signaling pathway [104]. Exosomes derived from ECFCs play a protective role against atherosclerotic or PTCA-induced EC damage by delivering miR-21-5p, which restores autophagic flux and inhibits SIAP1L2 expression [106]. Astragaloside IV promotes EPC-EXOs to regulate PI3KR2/SPRED1 signaling and inhibit apoptosis of diabetic ECs [135]. Moreover, miR-137 overexpression augments the neuroprotective effects of EPC-EXOs on hemoglobin-treated SH-SY5Y cells against apoptosis and mitochondrial dysfunction through the COX2/PGE2 signaling pathway [110]. Additionally, the combined presence of miR-126 in EPC-EXOs and miR-210 in neural progenitor cell-derived exosomes confers protection to neurons against apoptosis induced by hypoxia/reoxygenation through the Nox2/ROS and BDNF/TrkB pathways [136]. In addition, EPC-EVs can also exert direct cardioprotective effects by inhibiting cardiomyocytes apoptosis and hypertrophy. This is due to RNA carried by EPC-EVs can activate the PI3K/Akt/eNOS pathway [83]. Moreover, Yue et al. discovered that EPC-EXOs derived from wild-type mice had a significant positive impact on the cardiac function of the left ventricle after suffering AMI, effectively suppressing apoptosis in CMs and decreasing the size of the scar in the infarcted myocardium [117]. It can be seen that EPC-EVs have an irreplaceable role in inhibiting apoptosis.

### EPC-EVs and myocardial regeneration

The capacity of the human heart to regenerate cardiomyocytes is limited and decreases with age [137]. As individuals age, the number of CMs decreases gradually, leading to the inevitable development of heart failure since the primary reason behind it is the insufficient presence of CMs. The human left ventricle contains approximately 200–400 million cardiomyocytes, and AMI can eliminate around 25% of these cells in just a few hours [138]. Diseases that overload the heart, such as hypertension or valve disease, also slowly kill CMs [139]. Although it has long been believed that CMs are non-regenerative cells, more and more studies provide evidence that myocardial regeneration might occur in the hearts of newborn and adult mice [140–143]. In the past few years, the study of myocardial regeneration and repair has seen growing interest in EPC-EVs, which is mainly reflected in the following aspects: (1) Promoting CMs proliferation: EPC-EVs contain numerous growth factors, including VEGF, FGF, and insulin-like growth factor (IGF), etc. [26, 91, 119], These growth factors have the potential to directly stimulate the proliferation and differentiation of CMs, ultimately leading to an augmentation in the quantity of CMs [144–148]. (2) Inhibiting CMs apoptosis: EPC-EVs can hinder the expression of genes associated with apoptosis like bax and caspase-3, and attenuate CMs apoptosis by activating the signaling pathways, such as PI3K/Akt/eNOS [83]. (3) Promoting neovascularization: During phylogeny, a functioning vascular system is essential for a successful regenerative response [149]. After infarction, without neovascularization, the heart is unable to regenerate and ends up with extensive fibrous scarring [150]. The absence of neovascularization in adult mice with minimal regenerative response indicates that the inability to form new blood vessels after injury could result in a decline in the regenerative capacity of the heart [151, 152]. EPC-EVs contain numerous angiogenic factors. VEGF attaches to VEGFR located on the outer layer of ECs, stimulating the growth of ECs and the formation of new blood vessels [153]. Furthermore, the activation of platelet-derived growth factor (PDGF) signaling is essential for the growth of the epicardium and the development of neointima during the process of cardiac regeneration [154]. (4) Improvement of myocardial remodeling: Following AMI, alterations in the cardiac microenvironment are a vital part of the regenerative reaction, with the extracellular matrix (ECM) playing a critical function. Fibronectin facilitates the proliferation of CMs [155]. It has been found that the hearts of suckling rats repel fibrillated ECM deposits to the margins and promote myocardial regeneration in the weeks following myocardial injury [156]. EPC-EVs can improve myocardial remodeling by affecting the ECM after cardiac injury [114], which may be a mechanism that promotes the myocardial regeneration.

### Role of EPC-EVs in CVDs

**Fig. 2:**
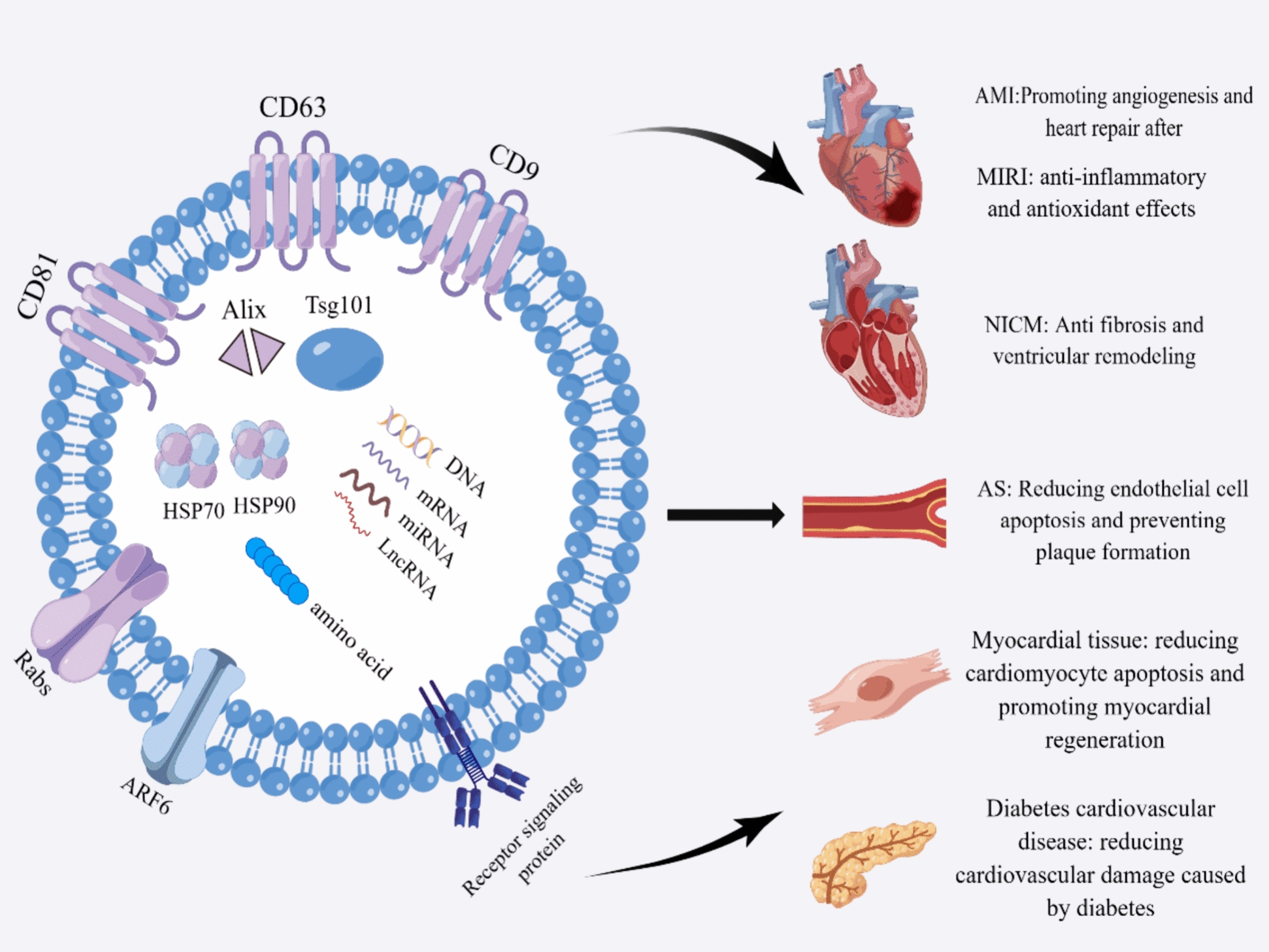
Biomarkers, bioactive substances and cardioprotective effects of EPC-EVs. EPC-EVs have typical markers such as tetraspanning membrane protein (CD9, CD63 and CD81), specific stress proteins (HSP70, HSP90), members of the ESCRT (Tsg101, Alix), proteins involved in membrane fusion (Rabs, ARF6) and signaling proteins. There are many biologically active substances present in EPC-EVs such as DNA, mRNAs, miRNAs, lncRNAs and proteins. due to these substances, EPC-EVs play many beneficial roles in CVDs. (CD, cluster differentiation; *HSP* heat shock proteins, *ESCRT* endosomal sorting complex required for transport, *CVDs* cardiovascular diseases, *AMI* acute myocardial infarction, *MIRI* myocardial ischemia–reperfusion injury, *NICM* nonischemic cardiomyopathy, *AS* atherosclerosis)

CVDs rank as the leading cause of morbidity worldwide, and are still the leading cause of premature deaths and rising healthcare costs in humans [157]. Therefore, the prevention and treatment of CVDs have become a global public health priority. EPC-EVs play an irreplaceable role in treating CVDs such as AMI, MIRI, atherosclerosis, nonischemic cardiomyopathy, and diabetic CVDs. Figure 2 shows the surface markers, active substances, and protective effects of EPC-EVs on CVDs.

## EPC-EVs and AMI

It has been shown that up to 10% of patients suffer cardiogenic shock immediately after AMI, with a 40-day mortality rate approaching 30% [158]. Despite the numerous clinical approaches available for the treatment and prevention of AMI, the irreversible ischemic demise of CMs persists, ultimately resulting in end-stage heart failure due to the substantial depletion of CMs following AMI. EPC-EVs contain a variety of active substances that promote angiogenesis and damage repair after AMI. For example, the enhancement of angiogenesis after AMI was mainly attributed to miR-1246 and miR-1290 in EPC-EXOs. These miRNAs targeted E74-like factor 5 (ELF5) and transcription factor Sp1 to regulate the phenotypic changes of fibroblasts into ECs, thereby exerting cardioprotective effects [159]. It has also been shown that IL-10-deficient EPC-EXOs upregulates ILK in exosomes, and ILK triggers NF-κB activation in the receiving cells to attenuate the therapeutic effect of EPC-EXO in infarcted myocardium, whereas knockdown of ILK in exosomes attenuated NF-κB activation and inflammatory response, suggesting that ILK is a key factor in the improvement of EPC-EXO-based cardiac therapy with target kinases [117]. In recent research, it has been shown that engineered EPC-EVs have improved therapeutic benefits for AMI. For example, using hydrogel microspheres containing engineered EPC-EVs activated by silicate has greatly enhanced angiogenesis in male mice following AMI. The reason behind this healing impact was credited to the presence of elevated levels of miR-126a-3p and angiogenesis factors (stromal cell-derived factor-1, VEGF, eNOS, and CD184) in the modified EVs. These EVs greatly enhanced hemodialysis, not only stimulating ECs but also attracting EPCs from the circulatory system [91]. Currently, engineered EPC-EVs have a promising future because they are easier to obtain, more therapeutically effective, and easier to convert into clinical drugs.

### EPC-EVs and MIRI

MIRI encompasses the detrimental consequences following the reopening of diseased vessels subsequent to AMI. During reperfusion, a surge in oxygen free radicals and calcium overload precipitates myocardial injury. The etiology of MIRI remains contentious, with prevailing theories implicating inflammatory responses, autophagy, apoptosis, calcium dysregulation, neurohumoral activation, and oxidative stress [160]. Evidently, EPC-EVs exhibit multifaceted properties including anti-inflammatory, antioxidant, and anti-apoptotic effects, suggesting their potential in mitigating MIRI. Notably, the macrophage-mediated inflammatory response assumes a pivotal role in MIRI [161]. miR-222-3p harbored within EPC-EXOs modulates macrophage polarization, fostering functional recovery in murine models post-Spinal Cord Injury via the SOCS3/JAK2/STAT3 pathway, underscoring the regulatory role of EPC-EXOs on macrophage phenotype [99].While the mechanistic insights into the action of EPC-EVs in MIRI are still emerging, initial investigations highlight their therapeutic promise. Studies have demonstrated that EPC-EVs bolster the viability and proliferation of umbilical vein ECs. Moreover, these EVs have shown promise in enhancing cardiac function post-MIRI in rats by fostering neovascularization while mitigating fibrosis and inflammation [162]. Furthermore, Ginsenoside Re augments the efficacy of EPC-EXOs in attenuating MIRI by orchestrating the miR-144-3p/SLC7A11-induced iron death pathway within exosomes [163].

### EPC-EVs and atherosclerosis

Atherosclerosis (AS) is a disease characterized by the formation of fibro-lipid plaques in the intima of arteries, leading to wall thickening and lumen narrowing. Both ECs and vascular smooth muscle cells (VSMCs) play key roles in this process. Damage to ECs is the initiating factor in the formation of AS, and VSMCs or macrophage phagocytosis of lipid-forming foam cells are the most important cells contributing to AS [164]. There is evidence that the transition of VSMCs from a contractile to a synthetic phenotype contributes to the progression of AS [165]. ACE2 overexpression by EPC-EXOs reduced VSMCs phenotypic changes by delivering functional ACE2 down-regulating NF-κB expression [166]. Abnormal EC-VSMC communication may lead to vascular wall remodeling and is strongly associated with the development of AS [167]. In Mature Vessels, VSMCs Connect with ECs by Activating BMPR2-Dependent Notch1 Signaling to Coordinate Chromatin Remodeling and Phenotypic Transformation of VSMCs to Enable ECs to Respond to Injury and Regenerate to Maintain Blood Flow and Vascular Homeostasis [168]. As the precursor cells of ECs, the research has shown that EPC is a potential controller of AS, and enhancing the function of EPCs can lower the risk of AS [169]. In addition, there are studies demonstrating that EPC-EVs can improve AS more significantly. For instance, Li et al. showed that EPC-EVs inhibited ECs iron death and delayed the onset of AS by inhibiting ECs iron accumulation, glutathione depletion, reactive oxygen species (ROS) production, and lipid peroxidation through miR-199a-3p/SP1 axis [109]. Similarly, it has been found that EPC-EXOs stimulates the growth, movement, and creation of blood vessels in human microvascular ECs(HMECs) by boosting the flow of autophagy and improving autophagic function as well as delivering miR-21-5p to inhibit SIPL1A2 expression, thereby preventing the development of AS and attenuating vascular injury [106]. In another study, EPC-EXOs overexpressing OCN promotes the vascularization of ECs and slows down the process of AS by enhancing OCN-GPRC6A signaling [120]. Furthermore, the administration of EPC-EXOs resulted in notable enhancement of endothelial function and decreased both plaque formation and the production of inflammatory factors in a mouse model of diabetic AS [98]. EPC-EXOs promoted an increase in ECs markers and a decrease in smooth muscle actin expression by inhibiting MEndT and decreasing HMGB1 expression [121]. In conclusion, damage and dysfunction of ECs, phenotypic transformation of VMSCs and foam cell formation promote AS, but EPC-EVs significantly inhibit the formation and development of AS by repairing damaged ECs and promoting the transformation of VMSCs from a synthetic to a contractile phenotype.

### EPC-EVs and nonischemic cardiomyopathy

Nonischemic cardiomyopathy (NICM) is a diverse collection of myocardial disorders with disturbances in the mechanical and electrical activity of the heart caused by different etiologies, accompanied by pathological changes such as inappropriate hypertrophy or dilatation of the ventricles, increased fibrosis, death of CMs, and impaired vascularization. NICM has become the primary reason for advanced heart failure and is responsible for over half of the total heart transplant cases [170]. EPC-EVs can ameliorate NICM through multiple mechanisms, such as inhibiting inflammatory response, suppressing fibrosis, and attenuating cardiomyocyte apoptosis. For example, EPC-EXO isolated from peripheral blood promoted MEndoT and decreased HMGB1 to inhibit cardiac fibroblast proliferation and promote angiogenesis in vitro [121]. In another research, it was demonstrated that the activation of the BRD4-mediated PI3K/AKT signaling pathway by miR-375-3p in EPC-EVs resulted in the reduction of inflammatory response, oxidative stress, apoptosis, and myocardial injury, leading to the improvement of septic cardiomyopathy in rats [171]. In general, EPC-EVs have potential applications in the treatment of NICM by promoting CMs repair, inhibiting fibrosis, promoting angiogenesis and improving microcirculation, anti-inflammatory, and immunomodulatory mechanisms. However, there is a lack of research on EPC-EVs and NICM, and further studies are needed to gain insight into their specific mechanisms of action and clinical application value.

### EPC-EVs and diabetic CVDs

Diabetes mellitus, a prevalent metabolic disorder, affects around 425 million adults globally at present. Additionally, diabetes-related fatalities and associated complications contribute to roughly 8.2% of total annual deaths [172]. As an independent risk factor for CVDs, diabetes mellitus causes microangiopathy. MIRI is more likely to occur with diabetes when the vessel is opened for reperfusion after an acute cardiovascular event, the degree of injury is further aggravated, and the incidence of adverse events like heart failure and cardiogenic shock during the later phase of the illness. The disease is significantly increased compared to patients with nondiabetic ischemic heart disease. The mechanism behind this correlation is believed to be closely associated with diabetic microangiopathy [173–175]. Moreover, individuals with diabetes experience a greater occurrence of AS, which manifests at an earlier age and advances more rapidly [176]. Diabetes inhibits the proliferation, migration, and angiogenic capacity of EPCs [177]. Hyperglycemic state alters the content and function of EPC-EVs [178], impairs the reparative function of EPC-EVs in ischemic cardiomyopathy, and inhibits angiogenesis in the ischemic heart [179]. However, EPC-EVs can mitigate the damage to the cardiovascular system by improving the functional impairment of ECs caused by diabetes. For instance, miR-126 and miR-296 in EPCs stimulate the growth and migration of islet ECs by activating the PI3K/Akt/eNOS signaling pathway, thereby promoting the formation of vascular-like structures. This process also plays a crucial role in maintaining the secretion of insulin by pancreatic β-cells, ultimately alleviating diabetes [180]. Moreover, the levels of angiogenesis-associated substances like VEGFA, FGF-1, angiopoietin-1, and E-selectin exhibited an elevation in ECs when exposed to the influence of EPC-EVs [26]. Furthermore, Zhang and his colleagues discovered that EPC-EXOs amplified the angiogenic function of ECs in a rat model with diabetes by activating the Erk1/2 signaling pathway, thus facilitating the healing and rejuvenation of skin wounds [126]. Another study found that the treatment of diabetic atherosclerotic (AS) mice with EPC-EXOs can significantly reduce plaque formation and the production of inflammatory factors by improving endothelial dysfunction, which is associated with the top 10 most highly expressed miRNAs in EPC-EXOs including miR-21a-5p, miR-222-3p, miR-221-3p, miR-155-5p and miR-29a-3p [98]. In summary, the contents of EPC-EVs, such as miRNAs, can mitigate the functional impairment of ECs in a diabetic environment, thereby alleviating diabetic cardiovascular diseases.

## Future directions and challenges

The potential for EPC-EVs to be widely used in treating CVDs is evident. First, EPC-EVs have multiple mechanisms of action in the treatment of CVDs, including promotion of cardiovascular regeneration, inhibition of inflammatory response, protection against CMs damage and antifibrosis [11, 95]. These mechanisms interact with each other and can target different aspects of CVDs in an integrated manner, thereby improving therapeutic efficacy and quality of survival. Second, EPC-EVs have good safety and tolerability. As a natural EV, EPC-EVs have low immunogenicity and toxicity, and are less likely to cause immune reactions and side effects [181]. In addition, EPC-EVs are easier to prepare and store, which facilitates mass production and clinical application [14]. Finally, the application of EPC-EVs also has better translational prospects and commercial value. By encapsulating EPC-EVs with specific nanomaterials or hydrogels, their retention time can be prolonged and localized sustained release can be achieved, greatly enhancing their therapeutic effect [182], Therapeutic VEGF-A mRNA transported by lipid nanoparticles can be delivered to other cells through the secretion of EVs, exerting a powerful therapeutic effect [183]. Furthermore, EPC-EVs can serve as drug delivery vectors for tissue regeneration and the treatment of various diseases, including ischemic stroke, myocardial infarction, diabetes, and acute kidney injury [184].

Currently, there are numerous studies on EPC-EVs in various fields such as CVDs, cancer, and immune diseases [185, 186]. Nevertheless, the utilization of EPC-EVs continues to encounter certain obstacles and issues. Firstly, the identification of biomarkers for EPCs remains contentious, compounded by their minimal presence in peripheral blood [90]. Culturing, isolating, and purifying EPCs is a complex, time-intensive, and financially burdensome process, exacerbating the overall cost of treatment and potentially impeding timely delivery of EPC-EVs. Secondly, optimizing the preparation and quality control of EPC-EVs is essential. This involves meticulous handling of various factors such as cell source, culture conditions, and stimulation methods to ensure the yield, purity, and functionality of EPC-EVs. Thirdly, the extraction and isolation of EPC-EVs necessitate the use of high-purity reagents and precise instrumentation, which can pose economic challenges. Moreover, there is a need for further in-depth exploration of the mechanism of action and biological properties of EPC-EVs. While numerous studies have demonstrated the multifaceted mechanisms of action and therapeutic effects of EPC-EVs, understanding the relationship and regulation between these mechanisms and effects requires additional investigation and clarification. Finally, despite abundant evidence from basic studies indicating the significant therapeutic potential of EPC-EVs, confirming their efficacy and safety is challenging due to the lack of extensive, long-term, and multicenter clinical trials.

In summary, EPC-EVs have broad application prospects, which can comprehensively treat multiple aspects of CVDs. It is worth noting that different injection methods produced different effects, with multiple localized injections within the myocardium being more effective than rat-tail vein injections, probably because the former is more likely to allow EVs to reach the site of injury. Despite some challenges and problems, there is a belief that EPC-EVs will emerge as a crucial therapeutic approach and strategic solution in addressing cardiovascular ailments, owing to the ongoing advancements in technology and the increasing depth of research. In the future, we can also look forward to the application of EPC-EVs in other fields, such as tumor therapy and immune disease treatment. Meanwhile, it is also necessary to continuously improve the related technology and clinical research to ensure the safety and efficacy of EPC-EVs and promote their wide application in clinical practice.

## Conclusion

EPCs, precursors to ECs, have attracted considerable attention in CVDs treatment due to their unique stem cell properties. Recent studies have underscored the potential of EPCs in vascular repair, neovascularization, and inflammation modulation, positioning them as promising candidates for CVDs therapy. EVs play pivotal roles in intercellular communication and have garnered increasing interest in diverse applications. Particularly, EPC-EVs have emerged as attractive cell-free therapeutic options in both basic research and clinical trials. Their small size, structural stability, low immunogenicity, and lack of infection risk render them favorable alternatives to EPCs.

EPC-EVs are enriched with various bioactive molecules, including miRNAs, lncRNAs, and proteins, endowing them with multifaceted therapeutic capabilities in CVDs such as AMI, MIRI, AS, NICM, and diabetic CVDs. Despite the promise of EPC-EVs, disparities in EPC sourcing, culture conditions, and identification processes across studies pose challenges to their quality and therapeutic outcomes. Additionally, the complex and resource-intensive nature of EVs extraction limits their widespread clinical adoption. Moreover, variations in EPC-EVs states necessitate precise control over preparation procedures for practical application. Addressing these challenges requires a deeper understanding of EPC cultivation, purification methods, EVs biogenesis, and diverse functions. Optimizing isolation processes and quality control measures for EPC-EVs will strengthen their viability in cellular-free CVDs therapy.

While large-scale clinical trial data on EPC-EVs in CVD treatment is limited, ongoing research into their mechanisms holds promise for advancing cellular-free therapies. Continuous optimization of EPC-EV preparation methods and exploration of their therapeutic mechanisms aim to offer safer and more effective treatment strategies for CVD patients in the future.

## Data Availability

Not applicable.
